# Risk Management Model for Unmanned Aerial Vehicles during Flight Operations

**DOI:** 10.3390/ma15072448

**Published:** 2022-03-26

**Authors:** Anna Kobaszyńska-Twardowska, Jędrzej Łukasiewicz, Piotr W. Sielicki

**Affiliations:** Faculty of Civil and Transport Engineering, Poznań University of Technology, Piotrowo 3, 60-965 Poznań, Poland; jedrzej.lukasiewicz@put.poznan.pl (J.Ł.); piotr.sielicki@put.poznan.pl (P.W.S.)

**Keywords:** air operation safety, flying risk, risk management, unmanned aerial vehicles

## Abstract

Risk management and uncertainty models are practised in all branches of transport. Although unmanned aerial vehicles (UAVs) constitute a branch of the industry rather than transport as a whole, their development is oriented toward increasingly more serious applications involving the transport of goods and people. The constantly growing number of operations employing UAVs requires not only identification of hazard sources or risk assessment recommended by the applicable regulations, but also comprehensive risk management. In order to develop a systematic approach to risk management for air operations of UAVs, the classic risk management method can be used. This work proposes a novel multi-criteria risk model that may serve as the basis for further activities aimed at developing a risk management method for this domain. The model was based on six criteria and validated using a virtual route to risk assessment and valuation.

## 1. Introduction

Due to their characteristics, the use of UAVs is increasingly common in industry, agriculture, construction, photography, and many other areas of human activity [1,2,3,4,5]. The use of UAVs in the energy production industry is also becoming more and more common. UAVs can be used, for example, to measure the amount of coal extracted in opencast mines, to study the composition of smoke emitted by power plants, and to monitor the technical condition of electricity transmission lines. In this context, the UAV is a platform for transporting the measuring device. Such a device can be an RGB camera, LIDAR, or an air quality measuring device.

**Figure 1:**
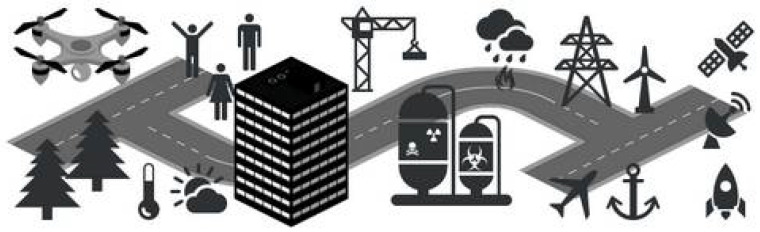
Application of UAVs and selected threads for consideration.

An example environment that includes selected threads during use of UAVs is shown in Figure 1.

Every transport system functions in conditions of uncertainty that threaten the accomplishment of its objectives related to infrastructure and transport organisation. Risk management is aimed at identifying events that may affect the accomplishment process [5]. Risk management has been practised in an unofficial manner for a very long time; events such as transportation, industrial, and economic disasters contributed to its systematisation, and then risks started to be dealt with in an organised and consistent manner in different areas of human activity [6]. This has led to the development and application of various methods, techniques, procedures, and tools classified under a common name: ‘risk management’ [7]. Many studies have examined risk management in chemical plants, nuclear power plants, and transport systems; this reflects that these areas generate a considerable number of hazards [7]. Generating hazards in various systems is one of the reasons why safety and risk management procedures were developed by entities involved in process execution in transport systems. Risk management models in transport systems can be found in studies [6,7,8,9,10,11,12,13,14,15,16,17], among others. Risk management should be treated as one of the tools of safety management systems [16]. Every entity managing elements of a transport system should also provide traffic safety management, ensuring observation and assessment of the number of accidents, casualties, and persons injured in accidents. Moreover, such entities should provide the possibility of completing a transport operation with the lowest risk possible. The path to such a state of safety leads to the development and skilful application of risk management methods [18,19,20]. Unmanned aerial vehicles (UAVs), i.e., multirotors, planes, and helicopters, are devices that—due to their functional characteristics—are used on an increasingly wide scale in different areas of human activity. Potential uses of UVAs were presented in [21,22,23].

The growing number of air operations that use UAVs entails a growing number of adverse events involving such vehicles. For this reason, work is underway to increase safety levels whilst operating UVAs. In study [24], the authors suggested a methodology for computing the probability of impact on 3D infrastructures, such as buildings, in the event of a UAV failure during flight. The generation of impact probability maps on the infrastructures is based on Monte Carlo simulations involving a dynamic model of a fixed-wing UAV. In another study, we find an integrated risk assessment method that considers probability and severity models of a UAV impacting people and vehicles on the ground. By introducing the gravity model, density of population and traffic are estimated on a finer scale, which enables more accurate risk assessment. The 3D risk-based path planning problem is first formulated as a special minimum-cost flow problem [25]. Study [26] proposes a framework for computing the risk of collision with an obstacle based on a UAV’s predicted trajectory, proximity to static and dynamic obstacles, sub-system state-of-health, and external wind conditions. The problem of safety in UAV operation was described in works [27,28,29,30], among others. In addition to scientific studies, there are also legal provisions that apply to UAVs, which are presented below.

The purpose of the article is to present a component of risk management for UAV flights, multi-criteria proposal, and a risk model developed based on a generalised risk model in the context of the applicable regulations.

## 2. Flight Categories and Assumed Risk Level

Within the European Union, flights of each UAV type take place based on the following EU regulations:

- Commission Delegated Regulation (EU) 2019/945 [31],
- Commission Delegated Regulation (EU) 2020/1058 [32],
- Commission Implementing Regulation (EU) 2019/947 [33].

These regulations define UAV classes and stipulate the rules and procedures related to the operation of these aircraft. According to [31,32], we distinguish seven UAV classes (C0–C6), depending on their equipment, weight, and forward speed in level flight. The categories on which the manner of risk management depends are [34]:

1. OPEN,
2. SPECIFIC,
3. CERTIFIED.

Air operations in the OPEN category may be conducted with an aircraft with maximum take-off mass (MTOM)—understood as the sum of the platform mass and the load mass—of less than 25 kg. The flight takes place within the visual line of sight (VLOS) and within 120 m from the closest point of the surface of the earth. The task of the remote pilot is to keep a safe distance from people [33].

The OPEN category is a non-significant or widely acceptable risk area that does not require risk-mitigating actions [33]. This is due to the low take-off mass, which does not exceed 250 g and 4 kg for subcategories A1 and A2, respectively. The low take-off mass generates low kinetic energy (e.g., for A1, E_K_ = 1/2 mv^2^) during an adverse event such as the UAV striking a person’s head. Moreover, in subcategory A2, flights may take place at a distance of 30 m from uninvolved persons or, in the case of low-speed-mode flights, at a distance of 5 m from them. Subcategory A3, which is also part of the OPEN category, is characterised by high take-off mass, but the pilot must conduct air operations over an area where it can reasonably be expected that, under normal circumstances, no uninvolved persons will be endangered, and at a distance of at least 150 m from residential, commercial, industrial, or recreational areas. According to the applicable regulations, the operator is not required to perform a risk assessment in this category of flights [33].

In the case of the SPECIFIC category, the risk related to the performance of the flight is tolerable, i.e., the transport aircraft may be operated, but under certain conditions [33]. This means that an authorisation is required for the performance of such flights, which must take place in accordance with the restrictions included in the operational authorisation or in the standard scenario defined by the legislative body. Flights may take place in compliance with other rules, provided that the UAV holds a light UAS operator certificate (LUC) with appropriate privileges. Authorisation to execute a mission can be obtained from the competent aviation authority in the given country [31]. In Poland, this authority is the Civil Aviation Authority. There are three ways of obtaining an operational authorisation. 

The first consists in the pilot making a declaration that they will conduct flights in compliance with the principles of conducting flights stipulated in the so-called standard scenarios. It is assumed that if the pilot conducts the flight in compliance with the principles defined in the standard scenario, the risk related to the performance of the flight is acceptable. Currently, two standard scenarios have been formulated within the European Union. Additionally, in Poland, so-called “national standard scenarios” apply, which are valid for flights conducted in VLOS and BVLOS (beyond visual line of sight), for aircraft masses of up to 4 kg and 25 kg, and for the following UAV types: multirotors, planes, or helicopters. The remote pilot has a total of eight scenarios, i.e., eight different variants of conducting the flight, at their disposal. 

The second method of obtaining the operational authorisation is to obtain the appropriate certificate (in the case of the EU, an LUC). The certificate is granted to the operator—understood to be a natural or legal person operating an aircraft—after they pass an inspection by the competent aviation authority in the given country. The certificate authorises the operator to make independent decisions about conducting a flight based on risk assessment. 

The third way of obtaining the authorisation, in the case of executing flight missions in a manner not described in the standard scenarios, is to submit a request to the competent aviation authority to issue the authorisation, along with the terms of conducting the flight based on a risk assessment performed independently by the operator. The currently recommended risk assessment method is that developed by the Joint Authorities for Rulemaking of Unmanned Systems (JARUS). The method is called the Specific Operations Risk Assessment (SORA) [34]. It is a very complex and time-consuming process that also requires access to a broad spectrum of technical information to which only the unmanned platform manufacturer has access.

The SORA method is a multi-stage method UAV flight risk assessment method. This assessment requires, inter alia: description of the concept of the operation (CONOPS), in which the drone operator, preparing for the mission, must describe all the details related to the flight, such as:

- List of UAVs used for the planned operation,
- Competences of the personnel involved in the operation,
- List of names of pilots and support staff,
- Procedures that will be applied in performing the mission,
- Indication of health requirements, which must be met by the personnel performing the mission,
- Declaration of the type of operation, including the purpose, method of performing the operation, restrictions due to environmental or legal conditions, scope of the operation (VLOS or BVLOS), indication of the area over which the operation will be performed together with the population density assessment, description of the so-called risk buffers, and description of the measures taken to ensure safety during the operation,
- Description of the aircraft technical data such as: aircraft size, mass, on-board equipment in the subsystem ensuring flight safety, limitations due to communication range or weather conditions, resistance to precipitation, conditions of minimum visibility, and conditions related to ambient temperature,
- Information regarding to the method of controlling UAV, including the method of controlling the ship and technical parameters of the Ground Control Station,
- Indication of the communication methods with air traffic control,
- Indication of the ways of avoiding collisions with other aircraft, including description of the systems used to achieve this, such as for example ADS-B,
- Description of the ground equipment and description of the fallback procedures.

The second step is to determine the intrinsic Ground Risk Class (GRC). This coefficient is determined on the basis of the assessment of the characteristic dimensions of the aircraft, such as its size, mass, and kinetic energy of potential collision with the ground. The next step is to define the so-called Final GRC of the impact hazard on the ground. In some cases, the GRC value, determined in step 2, may be so high that the resulting safety objectives to be achieved are too demanding for the operator. Therefore, to lower the GRC, one can either change the CONOPS or implement mitigation strategies. Consideration of measures, methods, and features of the system and mission that can positively affect the final GRC value can reduce the actual GRC value. For the reduction of GRC, for example, a parachute system can be used. The fourth step of the SORA assessment is the Determination of the Initial Air Risk Class (ARC). The airborne risk class depends on the determination of the chance of a collision with a manned aircraft. There are four classes, which can be distinguished from one with no risk of collision to one where the probability of collision is high. Another step in the SORA analysis is the application of measures at the strategic level and the definition of the end-risk ARC. Someone must use this step if the risk assessed in step 4th is too high. At this point, strategies, procedures, and constraints are applied to reduce the likelihood of a potential collision before the UAV takes off. The sixth step in the SORA analysis is the definition of the Tactical Mitigation Performance Requirement (TMPR) or the definition of Robustness Levels. In order to minimize the risk of an airborne collision with another aircraft, it is possible to apply tactical measures to reduce this risk. This stage defines the goals to be achieved at different levels of solidity so that a potential meeting in the air does not end in a collision. The seventh step is to organize the Final Specific Assurance and Integrity Levels (SAIL). The SAIL parameter consolidates the GRC risk with the ARC risk and allows to define the requirements for the operation. SAIL is a measure of the level of control over the security of a mission. SAIL is a requirement for a specific concept of operation. SAIL represents the level of confidence in the control of operations. The eighth step of the SORA assessment is the identification of safety objectives at the operational level; the so called Operational Safety Objectives (OSO). This step uses SAIL to assess the safety barriers and to determine their robustness. There are four grades of quality: optional, low robustness, medium robustness, and high robustness. The next step is to address the risk of losing control of the operation, resulting in the violation of adjacent areas on the ground and in the adjacent airspace. These areas may vary according to the different phases of flight. Accurately defining the adjacent area is the job of the operator. The adjacent area is assessed on the basis of whether the failure of the UAV could lead to the collapse of the UAV outside the operational area, assessment of the UAV systems in terms of their reliable maintenance of the UAV in the area of operation, or other threats, the activation of which may lead to the UAV’s escape outside the operational area. Once the assessments have been made in accordance with the procedure outlined above, the analysis document must be reviewed by the aviation authority, who can authorize the air operation. Regardless of the fact that this method is recommended by the Polish airspace authorities, the described level of complication of the SORA method and the evaluation model proposed by the authors described in the paper clearly show that the method proposed in the work is easier, does not require the assessment of so many parameters, and is much less time-consuming, which in the case of frequent unmanned missions is extremely important for the operator of aviation.

Article 11 of Regulation 2019/947 presents the procedure for risk assessment and allows for the development of a new, different operational risk assessment model. That model must include the following elements [33]:

- Description of the characteristics of the planned air operation,
- Description of the proposed adequate operational safety measures,
- Identification of the risks of operation on the ground and in the air,
- Risk-mitigating measures.

Flights in the CERTIFIED category occur with the use of UAVs certified based on Article 40 of Commission Delegated Regulation (EU) 2019/945. This category includes flights over assemblies of people, flights by UAVs designed for transporting people, and flights for transporting dangerous goods.

Regardless of the flight category, the risk level should be monitored. Control of the assessment area reduces the likelihood of the occurrence of adverse events (events which may lead to losses), but also facilitates rapid response if such an event does indeed occur. This is why the authors propose a risk model that makes it possible to assess the risk level for each type of flight.

In just the same manner as any other user of a transport system, a UAV operator should assess their physical and mental state before each air operation, and should also check and identify obstacles as well as potential sources of radio signal interference. Moreover, they should ensure an adequate safety level at the take-off and landing sites, including a reserve landing site. Therefore, from the point of view of safety engineering, the operator is responsible for assessing the risk [35]. The principles of the integrated risk management method based on the classical approach integrate two phases:

- Risk assessment, and,
- Responding to risk.

## 3. A New Risk Assessment Model

The nature of the organization and the goal it wants to achieve are factors that determine the choice of a risk management method. Within the framework of the classic risk management method, which the authors modelled, their components can be distinguished. There are two components in the risk assessment phase:

- Risk analysis,
- Risk evaluation.

The first component—risk analysis—is the systematic use of all available information in the indicated area of analysis, in order to:

- Identify threats—this is a process of systematic procedure to identify threats, which, as a result of their activation, may cause losses in the indicated area of analysis,
- Estimate and prioritize the risks identified in the analysis area—defining the value of the risk measure and assigning it to one of the risk levels of the model used.

As part of the risk assessment phase, the UAV operator should analyse the risk by characterizing the area and identifying potential hazard sources [33]. Next, they should assess the level of risk for the air operation by selecting the appropriate model and measures. The choice of the risk models and measures depends on the degree of complexity, detail, and the amount of information required and used [35,36,37,38,39]. In transport systems, the selection of the method depends on a number of factors, the scope of the process to be executed (e.g., transportation, infrastructure management, and maintenance), the availability of information on possible adverse events, and the experience of the people performing the assessment. An air operation must be preceded by an analysis of potential hazards that could lead to an air accident. There are five sources of potential hazards that, if activated, may cause a loss of control over the UAV, which may result in a UAV striking a person or object on the ground, or even another flying an unmanned or manned aircraft [39]. The five categories of hazard sources, as well as their respective contributing factors, are:

(1) Human error (i.e., human factors in aviation). This problem has been known and studied since the Second World War. Contributors to human error include:
  (a) Communication errors that could lead to a flight team not having full situational awareness;
  (b) Routine errors resulting from long-term aviation practice combined with loss of awareness of existing hazards, caused by frequently repeated activities;
  (c) Inappropriate or insufficient training of personnel;
  (d) Distraction resulting from disruption, confusion, or chaos, etc.;
  (e) Lack of team cooperation due to the lack of a sense of community purpose or communication style;
  (f) Fatigue caused by excessive working hours;
  (g) Lack of an appropriate tool to perform the task, i.e., inadequate aircraft to perform the planned mission;
  (h) Pressure from supervisors to fly in inappropriate conditions;
  (i) Insufficient assertiveness to refuse to perform a potentially hazardous task;
  (j) Stress caused by inadequate preparation for flight;
  (k) Carelessness, incorrect assessment of the situation, or incorrect assessment of the possible consequences of an air accident; and
(2) Failure to comply with procedures.
(3) UAS failure. Each aircraft is subject to pre-flight inspection in accordance with the procedures. However, each aircraft, as a technical facility, may fail during flight. The consequence of a failure in flight is almost always the fall of the craft to the ground.
(4) The appearance of another manned or unmanned aircraft on a collision course. Another aircraft may appear in the airspace in which flight operations are performed. An unexpected event may lead to errors in piloting and, consequently, to the fall of the aircraft.
(5) Rapid deterioration of weather conditions during the flight.
(6) Deterioration in the performance of systems used in steering or navigation, such as GPS. Solar activity is a source of high-intensity electromagnetic radiation emissions. The radiation emitted towards the Earth is usually absorbed by the atmosphere. Therefore, this radiation does not pose a threat to the operation of electronic systems used on board aircraft. However, if an electromagnetic pulse is too intense to be absorbed by the atmosphere, it will reach the Earth and may disrupt the operation of electronic systems. The consequence would be the loss of the ability to read the position of the aircraft from the navigation system, which may lead to an air accident. Electronic systems’ performance may also deteriorate as a result of flying in the vicinity of devices emitting electromagnetic radiation. Such devices include BTS stations used in mobile telephony and high-voltage lines.

**Figure 2:**
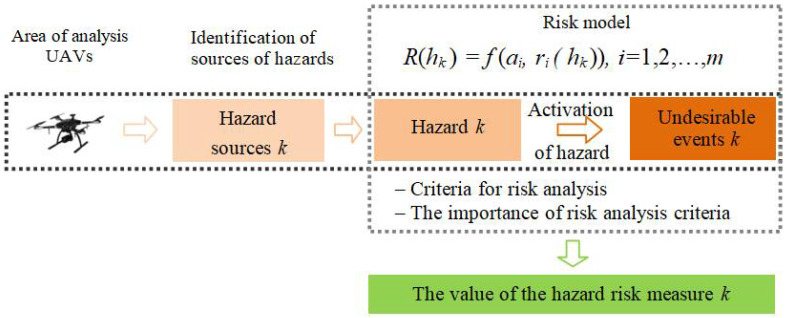
Place of the model and risk measures on the basis of [20].

The schematic diagram of the risk analysis and assessment using the developed model is presented in Figure 2.

In managing the risk of threats, there are detailed procedures, models, and risk measures dedicated to the areas of transport: road, rail, air, water, and urban. Currently, there is no model dedicated to the UAV transport system for risk assessment. Filling the research gap, an original risk assessment model for UVAs was proposed.

When developing the models and measures of hazard risks identified within the assessment areas, a generalised risk model presented in [36,37] may be adopted. 

Based on the generalised model presented in [38], for the assessment area of UAV flights in built-up areas, authors have developed the model for assessing risk levels. For the model, the set of hazards has the form (for this model, hazards are marked as *h*_1_, *h*_2_…*h_n_*):

*H_UAV_* = {*h*_1_, *h*_2_,..., *h_n_*} (1)

The risk model for each hazard from set *H_UAV_* is a function of components r_i_(*h_k_*) (*i* = 1, 2,..., *m*, *k* = 1, 2,..., *n*). Decisions are made based on the assessment according to 6 criteria *K_i_* (*i* = 1, 2, ..., 6) and the measures of significance a_i_ (*i* = 1, 2,..., 6) of these risk assessment criteria comprising the following set:

*A* = {*a*_1_,*a*_2_,...,*a*_6_} (2)

The importance measure for each criterion was defined with values from 1–6. In the risk model for UAV flights in a built-up area, 6 criteria with the following names and meanings were assumed:

K1: safety level criterion *SL*. The most important criterion *a*_1_ = 6. The measure of risk component *r*_1_(*h_k_*) according to this criterion is determined depending on the value of the safety level indicator (*SL*):

- Low when *SL* ≤ 4 × 10^−4^,
- Medium when 4 × 10^−4^ < *SL* < 2 × 10^−2^,
- High when *SL* ≥ 2 × 10^−2^.

The safety level indicator is expressed as follows:

*SL* = *L*_*I*_/*L*_*H*_ (3)

where:

*SL*—safety level indicator for UAV flights conducted in a built-up area,

*L_I_*—number of recorded incidents, and,

*L_H_*—number of flight hours logged.

The value of the *SL* indicator was determined based on analyses of the frequency of adverse events recorded by the entity performing the flight operations. The values of the *SL* indicator were presented in Table 1. In the analysis, the assumed annual flying time logged was 2688 h. The values included in the table were proposed based on the experience gained during remote pilot training conducted at the Poznań University of Technology.

K2: loss occurrence reach criterion. Criterion of importance measure *a*_2_ = 5. The criterion takes into consideration the type of material losses that may be caused by hazard activation. The losses concern the following subareas: infrastructure (subarea 1), natural environment (subarea 2), and people (subarea 3). According to this criterion, the measure of risk component *r*_2_*(h_k_)* is determined by the following principle:

- Low when the losses occurred in only one of the subareas,
- Medium when the losses occurred in two subareas, and,
- High when the losses occurred in all subareas.

K3: material loss criterion for material losses resulting from incidents involving UAVs. According to this criterion, the measure of risk component *r*_3_*(h_k_)* depends on the extent of material losses:

- Low when the losses do not exceed USD 2500,
- Medium when the material value of the losses exceeds USD 2500, but does not exceed USD 125,000, and,
- High when the value of material losses exceeds USD 125,000.

The values provided are based on the subjective assessment of the authors of the model. Measure of importance for this criterion was assumed at the level of *a*_3_ = 4.

K4: loss criterion based on the type of incident. Importance measure *a*_4_ = 3. The measure of risk component *r*_4_*(h_k_)* depends on the object with which the UAV collided:

- Low when UAV collides with another unmanned or manned aerial vehicle during flight,
- Medium when UAV collides with an obstacle on the ground, and,
- High when UAV collides with a person on the ground.

K5: hazard activation history criterion. Criterion of importance measure (*a*_5_ = 2). It is assumed that if a hazard was activated once, it is likely that it will be activated again. The measure of hazard activation *r*_5_*(h_k_)* is determined depending on hazard activation within a year preceding the assessment:

- Low when the event was recorded no more than 5 times,
- Medium when the event occurred no more than 10 times, and,
- High when the event occurred more than 10 times.

K6: hazard activation potential criterion. The criterion depends on the UAV type, competency certificates held, and flight location. The measure of risk component *r*_6_*(h_k_)* for this criterion is determined based on three elements (*K6.1, K6.2, K6.3*) characterising the flights performed.

Element *K6.1*—UAV type. This element of risk component *r*_6_*(h*_k_*)* indicates the potential for hazard activation depending on the UAV type (weight):

- Low when the UAV weight does not exceed 5 kg,
- Average when the UAV weight ranges between 5 and 25 kg, and,
- High when the UAV weight exceeds 25 kg.

Element *K6.2*—competency certificates. This element of risk component *r*_6_*(h_k_)* makes it possible to make the hazard activation potential dependent on the operator’s qualifications according to the following principle:

- Low if the operator holds a certificate with additional privileges,
- Average if the operator holds a certificate with basic privileges, and,
- High if the operator has no privileges.

In compliance with the provisions of the law, each remote pilot conducting a UAV flight is required to have formal qualifications and privileges for flights in the given category.

Element *K6.3*—distance from buildings. The measure of this element of risk component *r*_6_(*h_k_*) makes it possible to make the hazard activation potential dependent on the building density within the area where the flights are conducted:

- Low when there are no buildings within a 100-m radius,
- Average when there are buildings within a 30-m radius, and,
- High when there are buildings within a radius less than 30 m.

The measure of risk component *r*_6_(*z_k_*) is determined in accordance with the following principle:

- Low when a maximum of one element of criterion *K6* was rated as average,
- Medium when a maximum of two elements of criterion *K6* were rated as average, and,
- High when more than one element of criterion *K6* was rated as high.

Importance criterion *a*_6_ = 1.

The measures of risk component *r_i_*(*h_k_*) for each of the six risk model criteria for UAV flights assume the levels from set:

Ω = {low, medium, high}(4)

The elements of set Ω (Formula (4)) of the measures of risk components are assigned a set of risk measure values. Therefore, the result of risk calculation for each hazard from set *Z_UAV_* (Formula (1)), according to criterion *K_i_* (*i* = 1, 2..., 6) is the level of risk for component *r_i_*(*z_k_*) from the set of risk measure values. The function enabling estimation of the total risk measure taking into consideration the results of risk calculation according to the six criteria and significance measures of risk assessment criteria assumes the following form:

(5) $$R_{UAV}=\overset{6}{\underset{i=1}{\sum }}a_{i}\times r_{i}$$

The risk measures were selected subjectively following the principle of a starting wetness more important than the probability of their occurrence.

The next step should be risk evaluation, i.e., checking (by evaluation and by comparison) to which risk category (class) the estimated risk belongs (i.e., acceptable, tolerable, or unacceptable) [16]. 

The values of risk measures were determined, assuming an equal division for the adopted maximum and minimum.

The proposed risk acceptability classification for UAV operations was developed on the basis of the data from Table 2:

- Acceptable risk: no need to take actions to reduce the risk, but the control by the UAV operator should be below 49,
- Tolerable risk: the occurrence of personal or economic losses is medium or unlikely (the level of risk should be monitored); risk level is in the range of 49–78, and,
- Unacceptable risk: area where flight should not be allowed under any circumstances; risk level above 78.

The presented risk tolerability limits constitute only a proposal developed as a result of the work performed. Nevertheless, they can be shifted depending on the area in which BSP operations are performed and their nature.

## 4. Results

**Figure 3:**
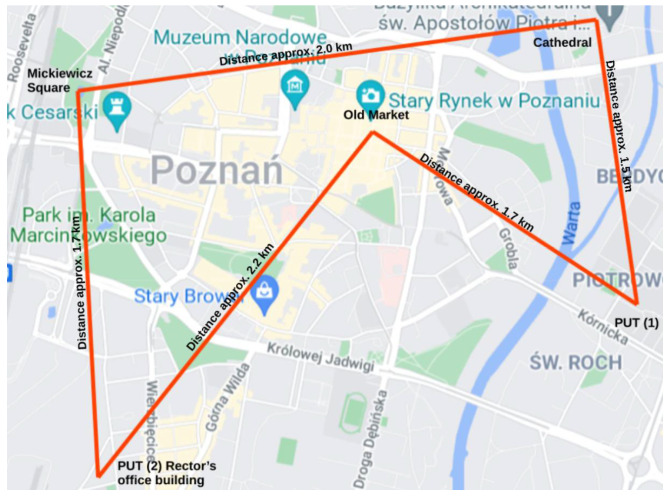
Flight route in Poznań [40].

The possibilities of using the model for the analysis areas related to drone operations located within Poznań were indicated. In order to show an example of an analysis using the model described in the work, a route between real points located in the Polish city of Poznań was proposed (Figure 3). The task that has been programmed before take-off on the drone’s computer is to take a photo at the locations indicated by the pilot. The purpose of taking pictures is to control the number of people in places popular among tourists. Such a control may be performed by the police, who need to know where to send patrols to maintain law and order. The flight occurs in conditions where there is no terrorist threat and in a situation where the state is not involved in an armed conflict. The drone does not carry any dangerous cargo, and the only useful cargo that has been mounted on the platform is a camera. The UAV will return to the place of take-off and land after completing the task. The UAV’s task will be to take photos in the places indicated by the pilot.

Assumptions:

1. The flight takes place at a fixed altitude above the rooftops of city buildings.
2. The cruising speed is 30 km/h, (8.3 m/s).
3. The ambient temperature is 24 degrees Celsius, the wind speed is 0 km/h.
4. LiPo battery works for 20 min.

Calculations: S = V × t, therefore we have about 9960 m for the cruise.

Route:

1. The flight begins in the parking lot of the Poznań University of Technology (PUT); the destination is the Old Market Square. Distance approx. 1.7 km.
  Threat sources:
  - Students,
  - PUT service personnel,
  - People passing on the street,
  - Cars,
  - Trees,
  - Cables disconnected in space,
  - Chimney on Grobla Street,
  - Buildings along the flight route,
  - Antennas on buildings.
2. Lowering the flight on Old Market Square to take a photo.
3. A flight from the Old Market Square to another location among the buildings of the PUT. Distance about 2.2 km.
  Threat sources:
  - People,
  - Buildings,
  - Trees,
  - Antennas on roofs,
  - The tower of the Church of Mary Queen in Wilda, approx. 45 m high,
  - Rector’s office building, approx. 40 m high,
4. Lowering the flight over the PUT parking lot in order to take a photo.
5. Flight from the car park in front of the PUT to the Imperial Castle on Św. Marcin street. Distance approx. 1.7 km.
  Threat sources:
  - People,
  - Buildings,
  - Trees,
  - Antennas on roofs.
6. Lowering the flight over Mickiewicz Square in order to take a photo.
7. Flight from Mickiewicz Square to the Cathedral. Distance approx. 2.0 km.
  Threat sources:
  - People,
  - Buildings,
  - Trees,
  - Antennas on roofs,
  - Okrąglak building,
  - Przemysl Castle.
8. Lowering the flight over the Cathedral in order to take a photo.
9. Flight from the Cathedral to the place of the drone take-off and landing. Distance 1.5 km.
  Sources of threats:
  - People,
  - Trees,
  - Cars.

The flight takes place at an altitude of 90 m above ground level. Lowering the altitude to take a photo means flight at an altitude of 20 m above ground level.

Risk assessments of threats identified on the flight route in Poznań have been achieved by following the principles of the risk model for UVAs, good engineering practice, and the knowledge of the authors. Table 3 shows risk assessment results for two selected threats.

## 5. Conclusions

The risk model indicates the algorithms and parameters of risk assessment and evaluation procedures. The original model is based on six criteria analysis with the possibility of taking into account the validity risk components obtained on the basis of each of the analysis criterion.

Meeting social expectations related to the operation of unmanned aerial vehicles largely depends on the effectiveness of risk management processes for hazards generated in this area of human activity. Currently, UAVs take off, fly up, and automatically cover their routes along the set flight paths. In order to operate without collisions, it is necessary not only to conduct a risk analysis, but also to introduce comprehensive risk management procedures—as in the case of all modes of transport—and the most important of these is to indicate the method or model of risk assessment.

Regulations recommend taking actions aimed at risk assessment. The methods developed for the purposes of such analysis for unmanned flights are far from sufficient.

The risk model presented in the paper is part of the risk management process. Risk estimation is preceded by the selection of the risk model and risk measurement model of threats identified in these analysed areas. The paper proposes a six-criteria risk model for unmanned aerial vehicles, which allows for the presentation of risk in a measurable manner. The criteria relate, inter alia, to the number of losses that may occur during the process being conducted in the human–technology–environment system, the probability of adverse events, and the history. Measures of criteria validity from 1–6 were adopted. For the criteria mentioned, qualitative measures of risk analysis were defined as low, medium, and high, which can be replaced by quantitative risk measures; for example, 1, 3, and 5 for this model. An exemplary UAV flight route in Poznań was developed and the risk for selected identified threats was estimated.

## Figures and Tables

**Table 1:** Safety level indicators.

Frequency	*SL*
1/1	1
1/8	0.125
1/56	0.018
1/224	0.0045
1/2688	0.0004

Source: Authors’ own elaboration.

**Table 2:** Classification of risk acceptability in UAV operations.

Criterion	Qualitative Measure	Quantitative Measure	Criterion Importance Measure	Risk Component Value	Total Level
*z_n_*—hazard when all measures of threat activation are low (minimum risk level)					
K1	low	1	6	6	21
K2	low	1	5	5	
K3	low	1	4	4	
K4	low	1	3	3	
K5	low	1	2	2	
K6	low	1	1	1	
*z_m_*—hazard when all measures of threat activation are high (maximum risk level)					
K1	high	5	6	30	105
K2	high	5	5	25	
K3	high	5	4	20	
K4	high	5	3	15	
K5	high	5	2	10	
K6	high	5	1	5	

Source: Authors’ own elaboration.

**Table 3:** Summary of the results of the risk assessment of selected threats generated during the flight in Poznań.

Criterion	Qualitative Measure	Quantitative Measure	Measure of Criterion Importance	Risk Component Value	Total Level	Risk
*z*_1_—possibility of hitting a car parked on the premises of PUT						
K1	low	1	6	6	35	acceptable
K2	low	1	5	5		
K3	low	1	4	4		
K4	medium	3	3	9		
K5	medium	3	2	6		
K6	high	5	1	5		
*z*_2_—possibility of loss of health in the event of a UVA impact						
K1	low	1	6	6	49	tolerable
K2	low	1	5	5		
K3	medium	3	4	12		
K4	high	5	3	15		
K5	medium	3	2	6		
K6	high	5	1	5		

Source: Authors’ own elaboration.

## Data Availability

Not applicable.

## References

[1] Alexis K., Nikolakopoulos G., Tzes A., Dritsas L., Valavanis K.P. (2009). Coordination of helicopter UAVs for aerial forest-fire surveillance. Applications of Intelligent Control to Engineering Systems.

[2] Goodarzi F.A., Lee D., Lee T. (2015). Geometric control of a quadrotor UAV transporting a payload connected via flexible cable. Int. J. Control Autom. Syst..

[3] Elmokadem T. (2019). Distributed coverage control of quadrotor multi-UAV systems for precision agriculture. IFAC-PapersOnLine.

[4] Rodríguez-Mata A.E., Flores G., Martínez-Vásquez A.H., Mora-Felix Z.D., Castro-Linares R., Amabilis-Sosa L.E. (2018). Discontinuous high-gain observer in a robust control UAV quadrotor: Real-time application for watershed monitoring. Math. Probl. Eng..

[5] Zieniuk K. Traffic safety at rail crossroads. Proceedings of the 2nd Polish Technical Conference.

[6] Jamroz K., Chruzik K., Gucma L., Kadziński A., Skorupski J., Szymanek A., Krystek R. (2009). Analiza możliwości integracji metod zarządzania ryzykiem podrozdział 7.3. Integrated Transport Safety System, Vol 2, Conditions of the Development of Integration of Transport Safety Systems.

[7] Szymanek A. (2012). Theory and Methodology of Risk Management in Traffic.

[8] Grisaro H.Y., Turygan S., Sielicki W.P. (2021). Concrete Slab Damage and Hazard from Close-In Detonation of Weaponized Commercial Unmanned Aerial Vehicles. J. Struct. Eng..

[9] Jamroz K. (2011). Risk Management Method in Road Engineering.

[10] Bonneson J., Mccoy P. (1997). Effect of Median Treatment on Urban Arterial Safety: An Accident Prediction Model. Transportation Research Record. J. Transp. Res. Board.

[11] Bureika G., Gaidamauska E., Kupinas J., Bogdevičius M., Steišūnas S. (2016). Modelling the assessment of traffic risk at level crossings of Lithuanian railways. Transport.

[12] Ford G., Heneker D. Australian Level Crossing Assessment Model. Proceedings of the Presentation to the International Rail Safety Conference.

[13] Kadziński A., Kobaszyńska-Twardowska A., Gill A. The Concept of Method and Models for Risk Management of Hazards Generated at Railway Crossings. Proceedings of the International Conference Transport Means.

[14] Lovegrove G.R., Sayed T. (2006). Macro-level collision prediction models for evaluating neighbourhood traffic safety. Can. J. Civ. Eng..

[15] Skorupski J., Gruszecki J. (2011). Methods of risk management in air traffic control. Selected Problems of Controlling Flying Objects.

[16] Skorupski J. (2018). Quantitative Methods of Air Traffic Incident Analysis.

[17] Jamroz K., Chruzik K., Gucma L., Kadziński A., Skorupski J., Szymanek A., Krystek R. (2010). The concept of the method of risk management in transport. The Integrated System of Transport Safety, Vol 3, Conditions for the Development of Transport Safety Systems Integration.

[18] Chi C.-F., Sigmund D., Astardi M. (2020). Classification Scheme for Root Cause and Failure Modes and Effects Analysis (FMEA) of Passenger Vehicle Recalls. Reliab. Eng. Syst. Saf..

[19] Pauer G., Török A. (2022). Introducing a novel safety assessment method through the example of a reduced complexity binary integer autonomous transport model. Reliab. Eng. Syst. Saf..

[20] Kadziński A., Gill A. (2011). The concept of implementation of the Trans-Risk method to risk management in tram transport. Logistyka.

[21] Aliyari M., Behooz A., Zewdu Ayele Y. (2021). Hazards identification and risk assessment for UAV-assisted bridge inspections. Struct. Infrastruct. Eng..

[22] Arravind R., Manjunath J., Midhunesh E., Tamilselvan D. (2021). Seed Dropping UAV: Design of Seed Dropping UAV in Afforestation. Int. Adv. Res. J. Sci. Eng. Technol..

[23] Kobaszyńska-Twardowska A., Łukasiewicz J., Scheffs W., Jaszczur W., Kamiński P. (2020). Monitoring State on Railway and Road Journeys with the Use of Unmanned Aircraft, Education in Safety and Defence.

[24] Levasseur B., Bertrand S. Impact Probability Maps Computation and Risk Analysis for3D Ground Infrastructures due to UAV Operations. Proceedings of the 2021 International Conference on Unmanned Aircraft Systems at Athens.

[25] Pang B., Hu X., Dai W., Low K.H. (2021). Third Party Risk Modelling and Assessment for Safe UAV Path Planning in Metropolitan. arxiv.

[26] Banerjee P., Gorospe G. Risk Assessment of Obstacle Collision for UAVs Under Off-nominal Conditions. Proceedings of the Annual Conference of the PHM Society.

[27] Jiang N., Wang K., Peng X., Yu X., Wang Q., Xing J., Li G., Zhao J., Guo G., Han Z. (2021). Anti-UAV: A Large Multi-Modal Benchmark for UAV Tracking. arxiv.

[28] Bigazzi L., Basso M., Boni E., Innocenti G., Pieraccini M. (2021). A Multilevel Architecture for Autonomous UAVs. Drones.

[29] Lopez B., Munoz J., Quevedo F., Monje C.A., Garrido S., Moreno E.L. (2021). Path Planning and Collision Risk Management Strategy for Multi-UVA Systems in 3D Environments. Drones.

[30] Prygies J. (2019). The UAVs Threat to Airport Security: Risk Analysis and Mitigation. J. Airl. Airpt. Manag..

[31] Commission Delegated Regulation (EU) 2019/945 of 12 March 2019 on Unmanned Aircraft Systems and on Third-Country Operators of Unmanned Aircraft Systems. https://eur-lex.europa.eu/legal-content/EN/TXT/?uri=CELEX:32019R0945.

[32] Commission Delegated Regulation (EU) 2020/1058 of 27 April 2020 Amending Delegated Regulation (EU) 2019/945 as Regards the Introduction of Two New Unmanned Aircraft Systems Classes. https://eur-lex.europa.eu/legal-content/EN/TXT/?uri=CELEX%3A32020R1058.

[33] Commission Implementing Regulation (EU) 2019/947 of 24 May 2019 on the Rules and Procedures for the Operation of Unmanned Aircraft. https://eur-lex.europa.eu/legal-content/EN/TXT/?uri=CELEX%3A32019R0947.

[34] Sora-Jarus. http://jarus-rpas.org/sites/jarus-rpas.org/files/jar_doc_06_jarus_sora_v2.0.pdf.

[35] Szopa T. (2009). Reliability and Safety.

[36] Chruzik K. (2016). Safety Engineering in Transportation.

[37] Sitarz M., Chruzik K., Wachnik A. (2012). Integrated safety management in rail transport. Integr. Manag. Syst. Saf. Eng..

[38] Kadziński A. (2013). Study of Selected Aspects of the Reliability of Systems and Facilities for Rail Vehicles.

[39] (2018). ULC Seminar on: Safety of UAV Flights, Warsaw.

[40] www.googlemaps.pl.

